# Elephant Seal Blubber Under Multiple Stressors: Persistent Organic Pollutants, Stress Hormones and Hydrostatic Pressure Alter Transcriptome, Lipolysis and Leptin Secretion

**DOI:** 10.1021/acs.est.6c05765

**Published:** 2026-08-31

**Authors:** Laura Pirard, Jane I. Khudyakov, Manon Martin, Louise Vanoost, Aurélien Warnant, Delphine Decloux, Thomas Baily, Daniel E. Crocker, Gauthier Eppe, Georges Scholl, Melissa M. Page, Jean-François Rees, Donald R. Smith, Cathy Debier

**Affiliations:** † Louvain Institute of Biomolecular Science and Technology, 83415 UCLouvain, Louvain-la-Neuve 1348, Belgium; ‡ Department of Biological Sciences, University of the Pacific, Stockton, California 95211, United States; § Department of Biology, Sonoma State University, Rohnert Park, California 94928, United States; ∥ Mass Spectrometry Laboratory, CART, MolSys Research Unit, 608565 University of Liège, Liège 4000, Belgium; ⊥ Microbiology and Environmental Toxicology Department, University of California Santa Cruz, Santa Cruz, California 95064, United States

**Keywords:** adipose tissue, cortisol, epinephrine, persistent organic pollutants, hydrostatic pressure, marine mammal, precision-cut slices, transcriptome

## Abstract

Anthropogenic stressors represent major threats to marine mammals. Yet, their combined effects on health remain poorly understood. To address this, precision-cut adipose tissue slices (PCATS) from weaned northern elephant seal (NES - *Mirounga angustirostris*) blubber were exposed to three treatments: (i) epinephrine (acute stress), (ii) cortisol, persistent organic pollutants (Aroclor 1254, BDE-47, BDE-99 and 4,4’-DDE) and variations of hydrostatic pressure mimicking NES diving conditions (chronic stress), and (iii) a combination of acute and chronic stressors. Compared to controls, acute, chronic and combined stressors altered the expression of 4854, 1544, and 8930 genes, respectively. Acute stress upregulated the expression of genes associated with inflammation and hypoxia and downregulated genes involved in lipid and xenobiotic metabolism. Chronic stressors activated inflammation, hypoxia and xenobiotic metabolism genes in exposed PCATS. Combined stress exerted the greatest impact on PCATS transcriptome, uniquely altering 4870 genes and regulating genes involved in lipid metabolism, inflammation and hypoxia. Epinephrine also stimulated PCATS lipolysis, while reducing leptin secretion. This study highlights how multiple stressors disrupt blubber metabolic functions, which are crucial for maintaining homeostasis during energy-demanding periods in marine mammals.

## Graphical abstract

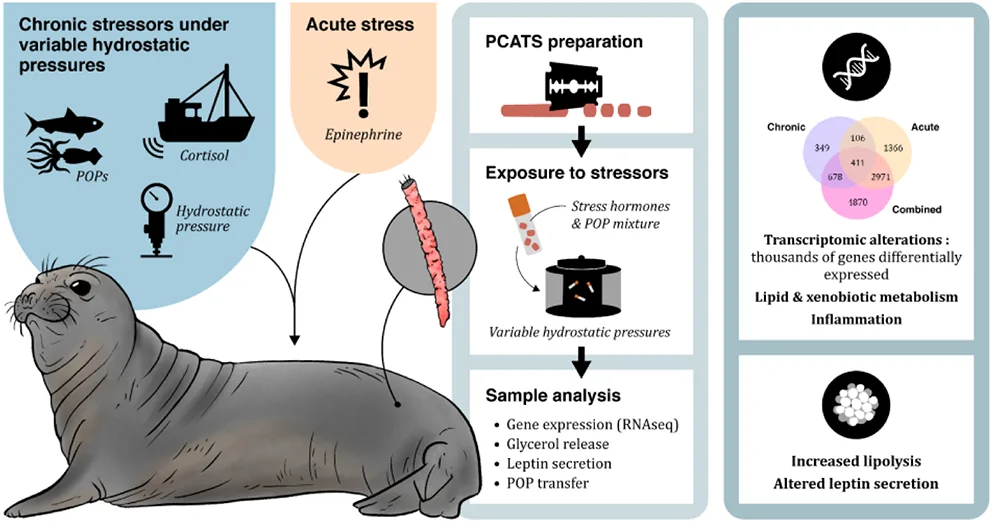

## 1 Introduction

Marine mammals face increasing anthropogenic stressors including acoustic disturbance, pollution, prey depletion and vessel-related injuries.^1,2^ These disturbances may (i) elicit stress responses in marine mammals, altering behavior and physiology^3–6^ and (ii) contribute to endocrine disruption, decreased immunity, lower reproductive success, and to population decline.^2,7–9^ Studying the cumulative impacts of multiple stressors *in vivo*, while crucial for conservation efforts, is challenging in free-ranging and federally protected species for practical and ethical reasons, necessitating the development of *ex vivo* models like precision-cut adipose tissue slices (PCATS).^10^

Blubber plays a crucial role in energy storage and endocrine regulation in marine mammals, making it a relevant target organ for investigating physiological stress responses. In addition to serving as the main energy reserve, blubber secretes signaling molecules (i.e., cytokines and adipokines). Among these, leptin is a hormone involved in the regulation of energy balance, food intake and neuroendocrine function.^11^ Through the secretion signaling molecules, blubber contributes to the regulation of metabolism, immune function, inflammation and reproduction.^12,13^ Additionally, it is the primary site for lipophilic persistent organic pollutant (POPs) accumulation, which is prevalent in marine mammal top predators.^14–16^ POPs are toxic, carcinogenic, and endocrine disruptors altering reproductive and immune functions.^17,18^ Blubber is also a target of stress hormones like catecholamines (e.g., epinephrine), rapidly stimulating lipid mobilization, and glucocorticoids (e.g., cortisol), contributing to sustaining energy availability.^19,20^ Prolonged elevation of glucocorticoid levels however can impair energy homeostasis, inflammatory response, reproduction, and lipid metabolism.^21^ Yet, their interaction with pollutants remains poorly understood.^22,23^

Studies of stress effects in marine mammals have been conducted at ambient pressure, whereas deep-diving animals routinely experience large fluctuations in hydrostatic pressures during diving.^24^ Pressure fluctuations affect membrane fluidity, transmembrane protein functions and molecule exchanges. Additionally, denaturation of proteins can be induced by hydrostatic pressure,^25^ which can have serious implications on redox homeostasis, potentially leading to oxidative stress and cellular damage. Animals exposed to hydrostatic pressures as part of their life histories have adapted by increasing the proportions of polyunsaturated fatty acids and cholesterol in membranes to enhance membrane fluidity and upregulating antioxidant defenses.^26^ To date, the effects of POPs and stress hormones on these adaptations remain unknown.

This study investigates the effect of stress hormones, POPs, and hydrostatic pressure on transcriptome and metabolic activity in marine mammal blubber. Using PCATS derived from northern elephant seals (NES),^10^ which accumulate POPs in their blubber^27^ and experience large changes in pressure during diving,^28,29^ we exposed tissues to three treatments. (i) We simulated an acute stress response by exposing PCATS to epinephrine, which is elevated in response to sympathetic activation (“acute stress”). (ii) We simulated chronic, multiple stressor exposure using cortisol and POPs under cyclic high-pressure conditions (“chronic stress”). This treatment was designed to mimic glucocorticoid elevation in response to hypothalamic-pituitary-adrenal axis activation in a tissue contaminated with POPs and exposed to fluctuating pressures during repeated diving – a combination of challenges that are realistically experienced by wild marine mammals. (iii) We used a combination of these treatments to examine the interactions between stress hormones – catecholamines and glucocorticoids – within the context of POP contamination and pressures experienced during deep diving (“combined treatment”). We analyzed transcriptomic response, POP accumulation, lipid metabolism (lipolysis and fatty acid profiles) and endocrine function (leptin secretion) in PCATS. This study addresses a critical knowledge gap by elucidating how acute and chronic stress interact to alter adipose tissue metabolism in deep-diving marine mammals.

## 2 Materials and Methods

### 2.1 Sample Collection and PCATS Preparation

All NES capture and handling procedures were approved by the Sonoma State University Institutional Animal Care and Use Committee (IACUC #2017–56) and were performed under National Marine Fisheries Service Marine Mammal Permit #23188. Blubber biopsies were collected from nine weaned male NES pups sampled early in their postweaning fast at Año Nuevo State Reserve, CA, United States (37°06′30’’N, 122°20′10’’W), between February and April 2022 (Table S1). PCATS and culture media were prepared as previously described.^10^ PCATS were then placed in 24-well plates filled with 1 mL of culture medium under agitation in an incubator (37 °C, 5% CO_2_). After 2 h, all culture media containing cellular debris caused by slicing were replaced with 1 mL of fresh media with appropriate treatments (i.e., stress hormones, POPs) as described below.

### 2.2 Stress Hormone Concentrations and Dilutions

The physiologically relevant media concentrations of 2 μM cortisol (Sigma-Aldrich) and 100 nM epinephrine (epinephrine bitartrate salt, Sigma-Aldrich) used for PCATS treatments were determined based on previous *in vivo* measurements in NES under physiological stress induced by exogenous adrenocorticotropic hormone (ACTH) injection^30^ as well as *ex vivo* experiments using NES PCATS.^23,31^ Cortisol was dissolved in absolute ethanol and diluted in water. The final concentration of ethanol in culture media was 0.01%. Epinephrine was dissolved in water.

### 2.3 POP Mixture Preparation

A mixture of Aroclor 1254 (DRE-C20125400, lot 1120968, Dr. Erenstorfer, LGC Group, Augsburg, Germany), BDE-47 (Dr. Erenstorfer), BDE-99 (Dr. Erenstorfer) and 4,4’-DDE (Sigma-Aldrich) was prepared. The pollutant proportions and concentrations used in our experiments were based on contaminant profiles reported in free-ranging marine mammal populations (Table S2), and consistent with concentration ranges commonly used in mechanistic *in vitro* experiments.^32–35^ As Aroclor 1254 is composed of a mixture of PCB congeners, the levels of seven indicator PCB congeners were measured in this mixture (Table S3). The proportion of Aroclor 1254 used in the pollutant mix was calculated based on PCB-153 concentration. A stock solution of each pollutant was prepared in dimethyl sulfoxide (DMSO; Sigma-Aldrich, Saint-Louis, United States), which is a solvent commonly used in cell culture experiments.^33,36,37^ PCATS were then exposed to the POP mixture for the entire culture period (45 h). The exact concentrations of POPs in the culture media were systematically quantified by gas chromatography prior to exposure to determine the actual exposure dose applied to PCATS. Concentrations in the culture media (mean ± SEM) were: 1.375 ± 0.342 μM 4,4’-DDE, 0.462 ± 0.071 μM PCB-153 (in Aroclor 1254), 0.038 ± 0.008 μM BDE-47 and 0.014 ± 0.003 μM BDE-99. DMSO was present in culture media at 0.036%, which is below the maximum recommended levels for cell culture.^38^

### 2.4 High-Pressure Cycle Treatment

To mimic hydrostatic pressures experienced by NES during routine dives, our laboratory adapted a hyperbaric system previously used to expose precision-cut fish liver slices to contaminants under high hydrostatic pressure.^39^ The hyperbaric system was composed of (i) a hyperbaric chamber (Autoclave, Rantigny, France) connected to an air-driven liquid pump (Maximator, Zorge, Germany) and a manometer used to control pressure levels, (ii) an atmospheric chamber maintained at ambient pressure as a control, and (iii) a water heater used for water circulation to maintain the temperature of both chambers at 37 °C (Figure S1). After 36 h of culture in a 24-well plate inside an incubator, PCATS were transferred into cryotubes (Corning, NY, USA) filled with preoxygenated culture medium. Filled cryotubes were placed in the hyperbaric chamber and high-pressure cycles were applied to PCATS as follows: hydrostatic pressure gradually fluctuated from atmospheric (1 bar) to high pressures (30 bar; equivalent to 300 m depth) at ascending and descending rates of 10 bar/min for a total of 20 min per high-pressure cycle, simulating pressures experienced by NES during routine dives.^24,28,40^ These variations of pressure were repeated six times, for a total of 2 h of high-pressure cycles. In parallel, cryotubes containing PCATS under the same experimental conditions were maintained at atmospheric pressure in the atmospheric chamber as controls (Figure S1).

### 2.5 Exposure of PCATS to Treatments

Three treatments were applied to PCATS obtained from 9 NES (Table S1): (i) acute stress, in culture media for 36 h, with 100 nM epinephrine applied for the last 9 h, between 36 and 45 h of culture, (ii) chronic stress, involving 2 μM cortisol and a mixture of POPs applied for 45 h, combined with six high-pressure cycles after 36 h of culture, for 2 h and, (iii) combined stress, integrating both acute and chronic stressors (Figure 1). PCATS cultured in parallel without hormones, POPs, or pressure treatment were used as a control (Figure 1). As the pollutant mixture was dissolved in DMSO, the same concentration of DMSO was added to control and acute stress treatments. PCATS were cultured with these treatments for 45 h and culture media were renewed after 12, 24, and 36 h in culture (Figure 1). At the end of the culture, media were collected and PCATS were rinsed twice in phosphate-buffered saline and flash-frozen in liquid nitrogen. PCATS and culture media were stored at −80 °C for further analyses. Samples dedicated to RNA sequencing were collected in RNase/DNA-free conditions.

**Figure 1:**
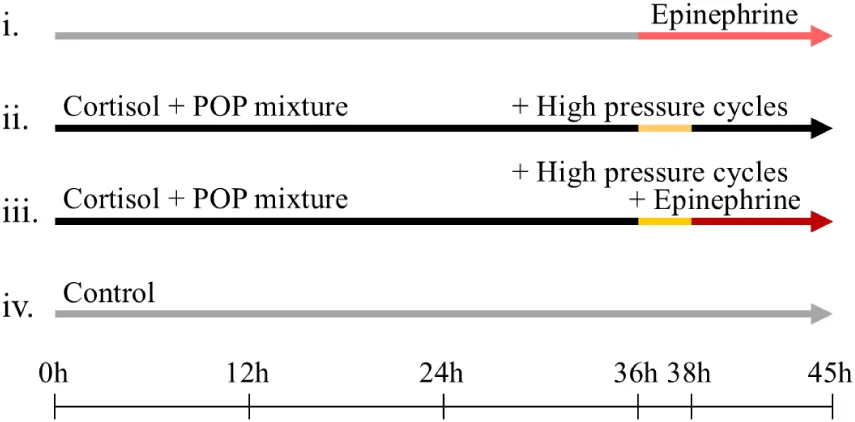
Experimental design illustration: Precision-cut adipose tissue slices (PCATS) from northern elephant seals were exposed to four treatments over 45 h of culture: (i) acute stress (epinephrine applied after 36 h), (ii) chronic stress (45 h-long exposure to cortisol and a mixture of persistent organic pollutants combined with 6 high-pressure cycles up to 30 bar lasting 20 min each after 36 h), (iii) combined stress treatment (acute combined to chronic stressors) and (iv) control (standard culture). Culture media were collected and replaced every 12 h.

A separate experiment examined the effects of cortisol and high-pressure cycles on POP accumulation in PCATS. Four conditions were compared: (i) control (no cortisol, atmospheric pressure), (ii) cortisol exposure (45 h, atmospheric pressure), (iii) high-pressure cycles (2 h between 36 and 38 h, no cortisol) and, (iv) combined treatment (cortisol exposure for 45 h and high-pressure cycles from 36 to 38 h). All conditions contained the POP mixture. This experiment was run using PCATS obtained from 6 NES pups (Table S1).

### 2.6 Viability

PCATS viability was assessed by measuring lactate dehydrogenase (LDH) released by damaged cells into culture media. Briefly, media were collected after the last 9 h of the 45-h culture and LDH level was measured using a cytotoxicity detection kit (Cytotoxicity Detection Kit (LDH), Roche, Sigma-Aldrich) and spectrophotometer (Spectramax 190 Microplate Reader, Molecular Devices). Values were normalized per g of tissue wet weight. There was no significant difference in LDH levels in media from control and treated PCATS from one individual pup (Table S1).

### 2.7 Quantification of POPs Transferred from Culture Media into PCATS

The transfer of POPs from the culture media into the PCATS samples was assessed using PCATS from six pups (Table S1). Baseline POP concentrations in blubber from these individuals were quantified prior to exposure. The analysis of seven indicator PCBs (PCB-28, 52, 101, 118, 138, 153 and 180), BDE-47, BDE-99 and 4,4’-DDE as well as the detailed calculation of POP transfer from culture media into PCATS are detailed in the Supporting Information.

### 2.8 Glycerol Quantification and Fatty Acid Composition of PCATS

Glycerol was measured in culture media collected after the last 9 h of culture by colorimetric assay (Glycerol assay kit, MAK117, Sigma-Aldrich) and using a Spectramax 190 Microplate Reader (Molecular Devices) following manufacturer’s protocol. Culture media from PCATS of six pups were used for this analysis (Table S1). For each pup, triplicates were analyzed. Values were normalized per g of tissue wet weight.

Analyses of the composition of PCATS fatty acid fractions were run using PCATS from three pups (Table S1) and are detailed in Supporting Information (Table S4).

### 2.9 Leptin Quantification

Leptin secretion from PCATS into culture media was measured using a radioimmunoassay according to the manufacturer’s protocol (Multispecies leptin kit, Linco, St. Charles, United States), which has previously been validated for NES.^41^ Culture media from PCATS of five pups were used for this analysis (Table S1). Concentrations of leptin were normalized per g of tissue wet weight.

### 2.10 RNA Isolation and Sequencing

PCATS from five pups were used for transcriptome analyses (Table S1). RNA samples had a mean (± s.d.) RNA integrity number of 8.4 ± 0.5. Detailed RNA isolation and sequencing procedures are available in Supporting Information.

### 2.11 Transcriptomic Analysis

Sequence quality was assessed with FastQC v0.11.9, and the transcriptome was assembled de novo using Trinity v2.11.0. Redundancy was reduced with MMSeqs2 (1,629,424 to 1,045,492 transcripts), and assembly completeness was evaluated with BUSCO v5.0.0 (Mammalia database; 82.2% complete). Transcript abundance was estimated with Salmon v1.9.0, and transcripts were annotated by BLASTX (DIAMOND v2.0.13) against UniProt SwissProt Mammalia/Caniformia databases, with unannotated transcripts further searched by BLASTN against RefSeq database. Details are available in Supporting Information. The transcriptome assembly files (Supplementary Files S1–S7) are available at: https://doi.org/10.14428/DVN/TOYCCT.

### 2.12 Statistical Analysis

Statistical analyses and visualizations of transcriptome data were conducted using R (v4.3.1) and R Studio (v2023.06.1). All supplementary files are accessible at https://doi.org/10.14428/DVN/TOYCCT. Differential gene expression analyses were conducted using the R packages “tximport” and DESeq2 v1.35.0 with individual NES ID from which blubber biopsies for PCATS were obtained as a blocking factor (formula = ∼ individual + treatment). Differentially Expressed Genes (DEG) were extracted with the significance cutoff alpha = 0.05 and the Benjamini-Hochberg correction for p-values (Supplementary Files S8–S10). Principal Component Analysis (PCA) plot of TMM-normalized and transformed (“vst” function in DESeq2) gene expression data was created using the “plotPCA” function in DESeq2. Gene set enrichment analysis (GSEA) was conducted using the “clusterProfiler” R package against the human genome background using the “Hallmark” gene collection of the Human Molecular Signatures Database (Supplementary Files S11–S13). Biological processes were considered significantly enriched at Benjamini-Hochberg adjusted (p adj.) *p* < 0.1. Dot plots of regulated biological pathways were generated using the “enrichplot” R package. Differences in glycerol, free fatty acid, leptin and LDH release as well as POP transfer between treatments were assessed using JMP PRO 16.0 software. PCATS responses to treatments for these outcomes were evaluated using linear mixed models, with treatment as a fixed effect and individuals from which PCATS were obtained as a random effect. Model assumptions (normal distribution of residuals and homoscedasticity of groups) were verified using model diagnostic plots. Glycerol and leptin concentrations were transformed by square root and logarithmic transformations, respectively, to meet the assumptions of the models. Student’s *t* tests with a Šidák correction were used to compare results between treatments.^44^ Šidák method is similar to Bonferroni’s correction, but less conservative and more accurate.^45^ The level of statistical significance was set at *p* < 0.05 for all analyses.

## 3 Results

### 3.1 Transcriptomic Response of PCATS to Multiple Stressors

Transcriptome assembly and clustering generated 1,045,492 Trinity transcripts in 1,006,066 transcript families, with an average contig length of 787 bp and contig N50 of 1145 bp. Of these, 168,783 and 352,621 transcripts had hits to proteins in the UniProt Mammalia and Caniformia databases, respectively. The transcriptome assembly contained 82.2% of highly conserved mammalian orthologs (BUSCOs), suggesting high completeness. Differential gene expression analysis of PCATS exposed to acute, chronic and combined stressors suggests a combinatorial effect of stress treatments on the blubber transcriptome. Principal Component Analysis (PCA) of global transcriptome profiles showed that samples clustered preferentially by treatment applied to PCATS (Figure 2) along the first two Principal Components. Samples exposed to acute and combined treatments were more closely related as compared to control and chronic stress conditions. However, there was a high variability in gene expression between PCATS obtained from different individuals.

**Figure 2:**
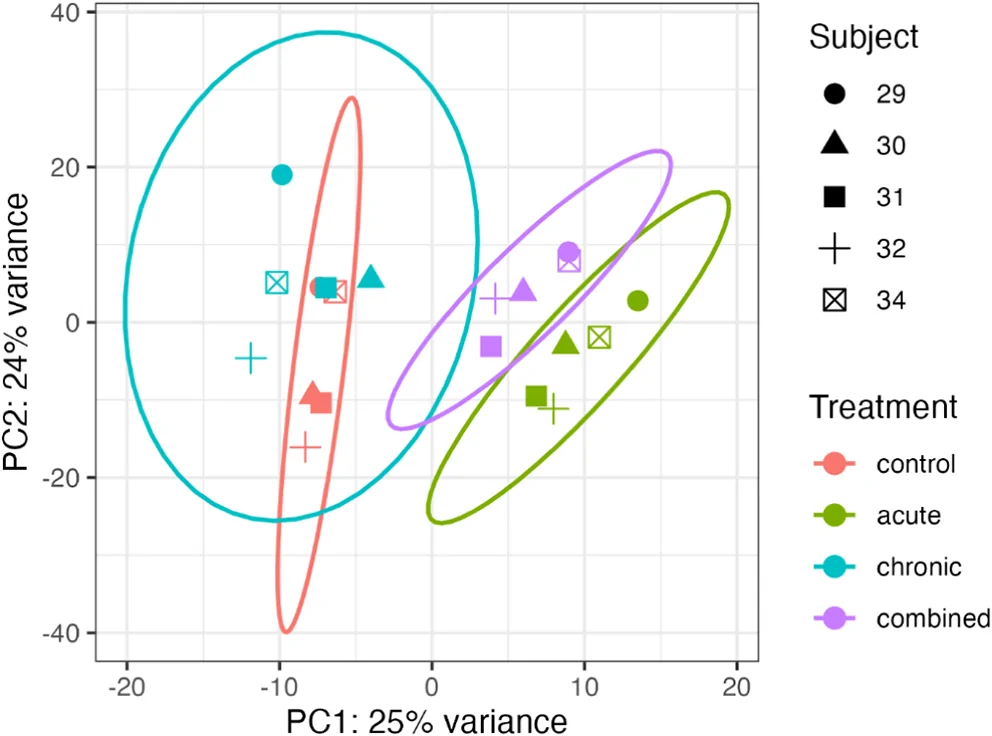
Principal component analysis (PCA) plot of transcriptomic data obtained from precision-cut adipose tissue slices (PCATS) from northern elephant seals (*n* = 5 male pups) exposed to multiple stress treatments: (i) acute stress (epinephrine applied after 36 h), (ii) chronic stress (45 h-long exposure to cortisol and a mixture of persistent organic pollutants combined with 6 high-pressure cycles up to 30 bar lasting 20 min each after 36 h), (iii) combined stress treatment (acute combined to chronic stressors) and (iv) control (standard culture). Symbol shapes show different individuals. Colors and 95% confidence ellipses denote treatments.

Relative to the controls, acute, chronic and combined stress treatments altered the expression of 4854 genes (2178 up-regulated and 2676 down-regulated), 1544 genes (1044 up-regulated and 500 down-regulated) and 8930 genes (4334 up-regulated and 4596 down-regulated), respectively (Supplementary Files S8–S10). Among these differently expressed genes, the combined stress treatment uniquely upregulated 2368 and downregulated 2504 genes that were not altered by any other treatment. Acute stress uniquely upregulated 702 and downregulated 781 genes and chronic stress upregulated 416 and downregulated 51 genes relative to control (Figure 3a). The heatmap representing the top 500 genes differentially expressed under acute treatment relative to the control, with all treatments represented, highlights similar patterns of gene regulation in PCATS exposed to acute and combined stressors, as well as in control and chronically stressed PCATS (Figure 3b).

**Figure 3:**
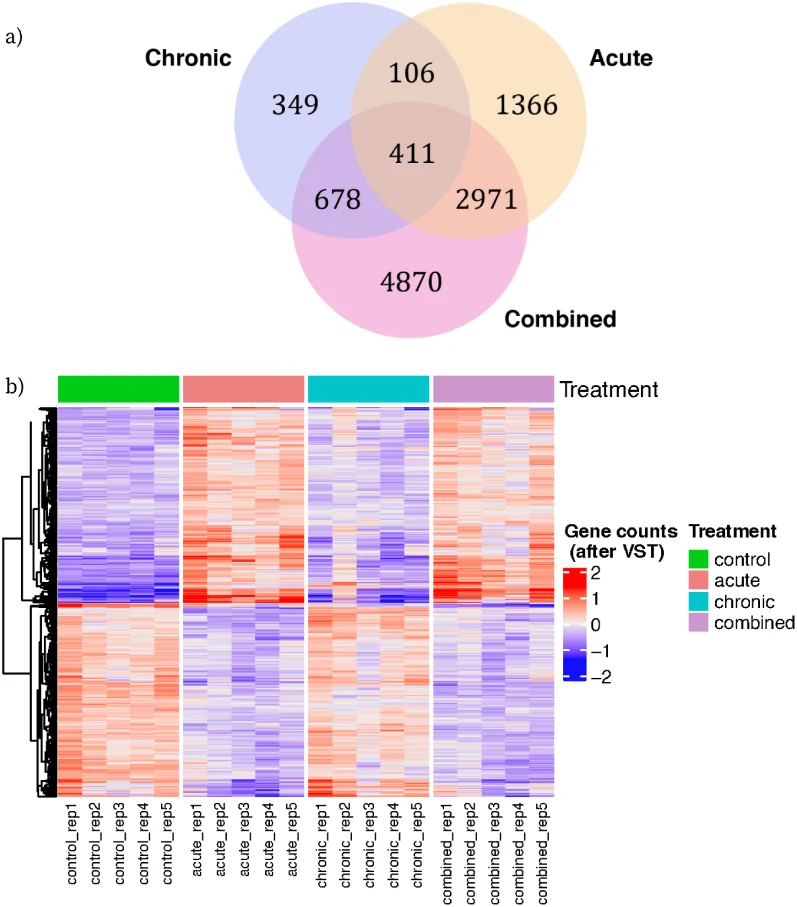
(a) Venn diagram showing the numbers of genes differentially expressed in PCATS from northern elephant seals (*n* = 5 male pups) in response to three experimental conditions relative to control: (i) acute stress (epinephrine applied after 36 h), (ii) chronic stress (45 h-long exposure to cortisol and a mixture of persistent organic pollutants combined with 6 high-pressure cycles up to 30 bar lasting 20 min each after 36 h), and (iii) combined stress treatment (acute combined to chronic stressors). (b) Heatmap of the 500 top genes differentially expressed in acute treatment relative to control. Results from five early weaned male northern elephant seal pups are shown. Colors represent scaled gene counts in PCATS from each experimental condition after variance stabilizing transformation (VST).

Gene set enrichment analysis (GSEA) identified a total of 14 enriched pathways in PCATS exposed to the acute treatment relative to control (Supplementary File S11). The five most significantly activated pathways included hypoxia, inflammation (TNFα signaling via NFκB), unfolded protein response and regulation of cell cycle (PI3K AKT MTOR signaling) (Figure 4a). The significantly suppressed pathways referred to peroxisomes, lipid metabolism (adipogenesis, bile acid metabolism and fatty acid metabolism), and xenobiotic metabolism (Figure 4a).

**Figure 4:**
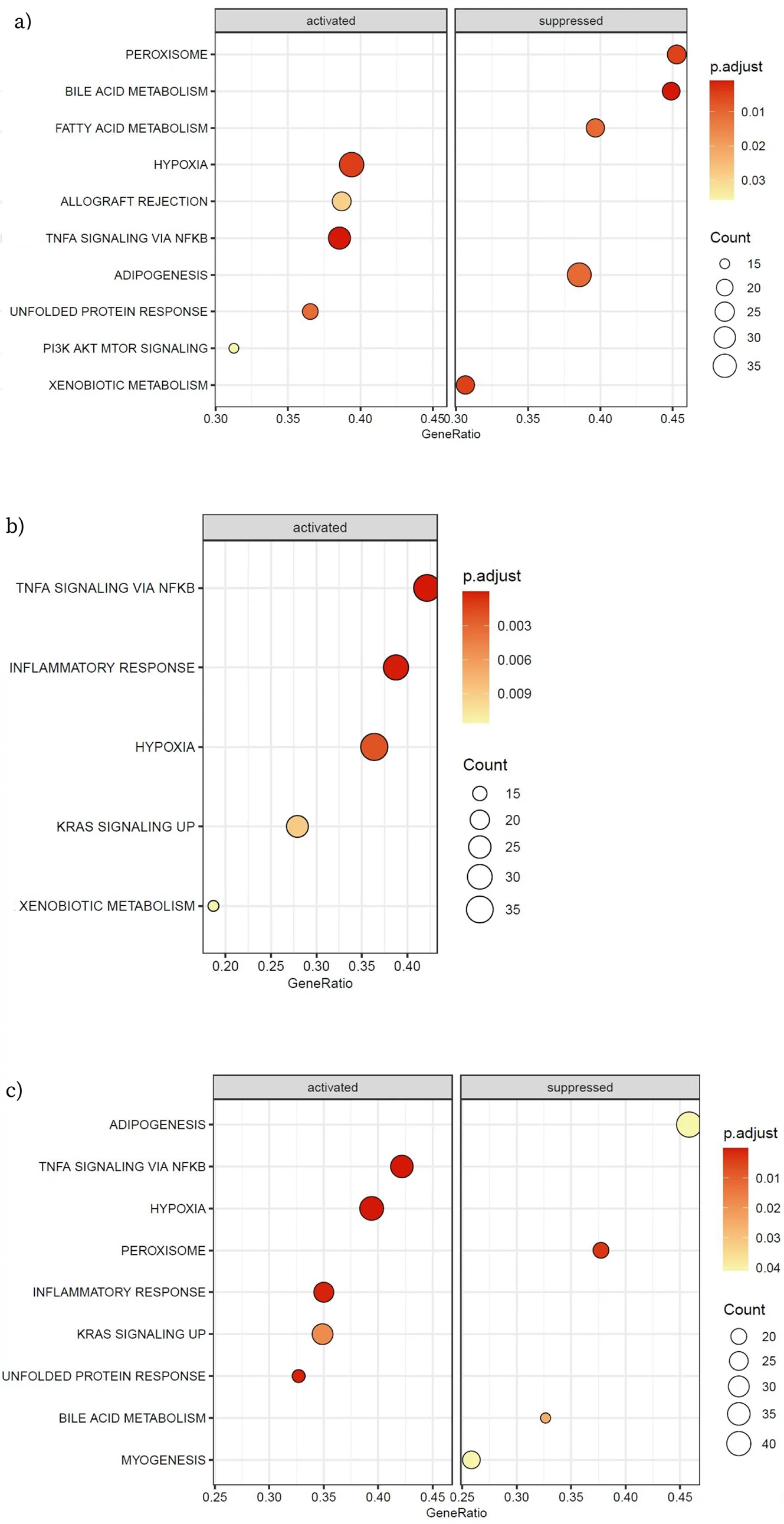
Top 5 of the most significantly enriched pathways (GSEA analysis, adj. *p* < 0.1) within sets of up- and down-regulated DEGs in stress-exposed PCATS from northern elephant seals (*n* = 5 male pups) relative to control. PCATS were exposed to a) acute stress (epinephrine applied after 36 h), b) chronic stress (45 h-long exposure to cortisol and a mixture of persistent organic pollutants combined with 6 high-pressure cycles up to 30 bar lasting 20 min each after 36 h), c) combined stress treatment (acute combined to chronic stressors). “Count” indicates the number of DEGs mapping to each biological process.

GSEA identified a total of 5 enriched pathways in PCATS exposed to chronic stress. They were related to cell growth (upregulation of Kirsten rat sarcoma virus oncogene homologue (KRAS) signaling), inflammation (inflammatory response and TNFα signaling via NFκB), hypoxia and xenobiotic metabolism (Figure 4b) (Supplementary File S12). There were no significantly enriched pathways within DEG that were downregulated by chronic stress treatment.

A total of 20 pathways were enriched in PCATS after exposure to the combination of acute and chronic treatments (Supplementary File S13). The five most enriched pathways are represented in Figure 4c. Activated pathways included cell growth (upregulation of KRAS signaling), hypoxia, inflammation (inflammatory response, TNFα signaling via NFκB) and unfolded protein response. Suppressed pathways corresponded to adipogenesis, bile acid metabolism, myogenesis and peroxisome.

Given that genes associated with lipid metabolism and related processes were differentially regulated in stress-exposed PCATS, a targeted screening of associated genes was performed (Table 1). Target genes were selected based on previous studies investigating physiological changes in elephant seal blubber,^46,47^ transcriptomic response of blubber to stress,^23,30^ as well as studies describing key regulators of lipid metabolism in mammalian model species.^48–51^ Stress-regulated genes encoding adipokines, cytokines and detoxification metabolism were also specifically analyzed (Table 1).

**Table 1:** Genes and Their Encoded Protein Products Related to Lipid Metabolism, Endocrine Function and Xenobiotic Metabolism That Were Differentially Expressed in PCATS from Northern Elephant Seals (N = 5 Male Pups) After Exposure to Stress Treatments Relative to Control^a^

Lipid-related process	Genes	Associated protein	Acute stress	Chronic stress	Combined stress
Acyl CoA synthesis	*ACSL1*	Long-chain-fatty-acid-CoA ligase (ACSL)	Down	Up	Down
	*ACSL3*	short-chain acyl-CoA synthase (ACSS)	=	=	Up
	*ACSL4*		Up	Up	Up
	*ACSL5*		=	Up	=
	*ACSS3*		down	=	down
Adipogenesis	*PPARG*	Peroxisome proliferator-activated receptor gamma (PPARγ)	down	=	down
Lipid droplet structure	*PLIN1*	Perilipin (PLIN)	up	up	up
	*PLIN2*		up	up	up
	*PLIN3*		=	=	up
	*PLIN4*		down	=	down
Lipid elongation and desaturation	*ELOV2*	Very long chain fatty acid elongase (ELOV)	=	=	down
	*FADS1*	Acyl-CoA desaturase (FADS)	down	down	down
	*FADS2*		down	down	down
	*FADS3*		=	=	down
	*FADS6*		down	=	down
Lipid hydrolysis	*MGLL*	Monoglyceride lipase	down	=	down
	*ADRB2*	β-adrenergic receptors	up	up	up
	*ADRB3*		up	=	up
Lipid synthesis	*GPD1L*	Glycerol-3-phosphate dehydrogenase 1-like	down	=	down
	*DGAT1*	Diacylglycerol acyltransferase 1	down	down	down
Phosphatidylinositol synthesis	*PI3R5*	Phosphoinositide kinase regulatory subunit Phosphatidylinositol kinase α	up	up	up
	*PI4KA*	Phosphatidylinositol kinase β	up	=	up
	*PI42A*		up	=	up
	*PI51A*		up	=	up
	*PI4KB*	Phosphatidylinositol 4-phosphate 5-kinase-like protein 1	down	=	down
	*PI42B*		up	=	up
	*PI5L1*		up	=	up
	*PI5PA*	Phosphatidylinositol 4,5-bisphosphate 5-phosphatase A	=	=	up
Endocrine function	*LEP*	Leptin	down	up	down
	*ADIPOQ*	Adiponectin	down	=	down
	*NAMPT*	Visfatin	=	=	up
	*IL6*	Interleukin 6	up	=	=
Xenobiotic metabolism	*CYP1A1*	Cytochrome P450	down	up	up
	*CYP2W1*		up	=	up

a PCATS Were Exposed to (I) Acute Stress (Epinephrine Applied After 36 H), (Ii) Chronic Stress (45 H-Long Exposure to Cortisol and a Mixture of Persistent Organic Pollutants Combined with 6 High-Pressure Cycles Up to 30 Bar Lasting 20 Min Each After 36 H), (Iii) Combined Stress Treatment (Acute Combined to Chronic Stressors) and (Iv) Control (Standard Culture)

Most genes involved in adipogenesis and fatty acid synthesis, elongation and desaturation were downregulated while those related to lipid droplet structure and β-adrenergic receptors were upregulated in all stress treatments (Table 1). *LEP*, encoding leptin, was downregulated following acute and combined stress exposure but upregulated under chronic stress conditions. *IL6* was specifically upregulated in response to acute stress. In contrast, *CYP1A1* was downregulated by acute stress and upregulated by chronic and combined stressors.

### 3.2 Stress Impact on POP Accumulation in PCATS

The transfer of POPs into PCATS correlated with their concentration in the culture media, as indicated by a high coefficient of determination of linear regression (coefficient of determination R^2^ = 0.94) (Figure 5a). When all pollutants were considered in the analysis, exposure to cortisol did not affect the transfer of POPs from culture media into PCATS (*p* > 0.05). On the other hand, high-pressure cycles significantly decreased their incorporation into tissues (*p* < 0.05; Figure 5b). While high pressure slightly altered the transfer of total POPs, it did not affect concentrations of individual pollutants in PCATS. POPs accumulated in PCATS in a dose-dependent manner, therefore the constituents of the exposure cocktail that were present in higher concentrations in the culture medium were also detected in higher concentrations in PCATS. The initial concentration of POPs in NES blubber was analyzed and showed similar levels of contaminants in all but one individual that had concentrations 3 times lower than the other pups (data not shown). The initial contamination of animals accounted for 2% of the contamination after 45 h of exposure to the POP mixture (data not shown).

**Figure 5:**
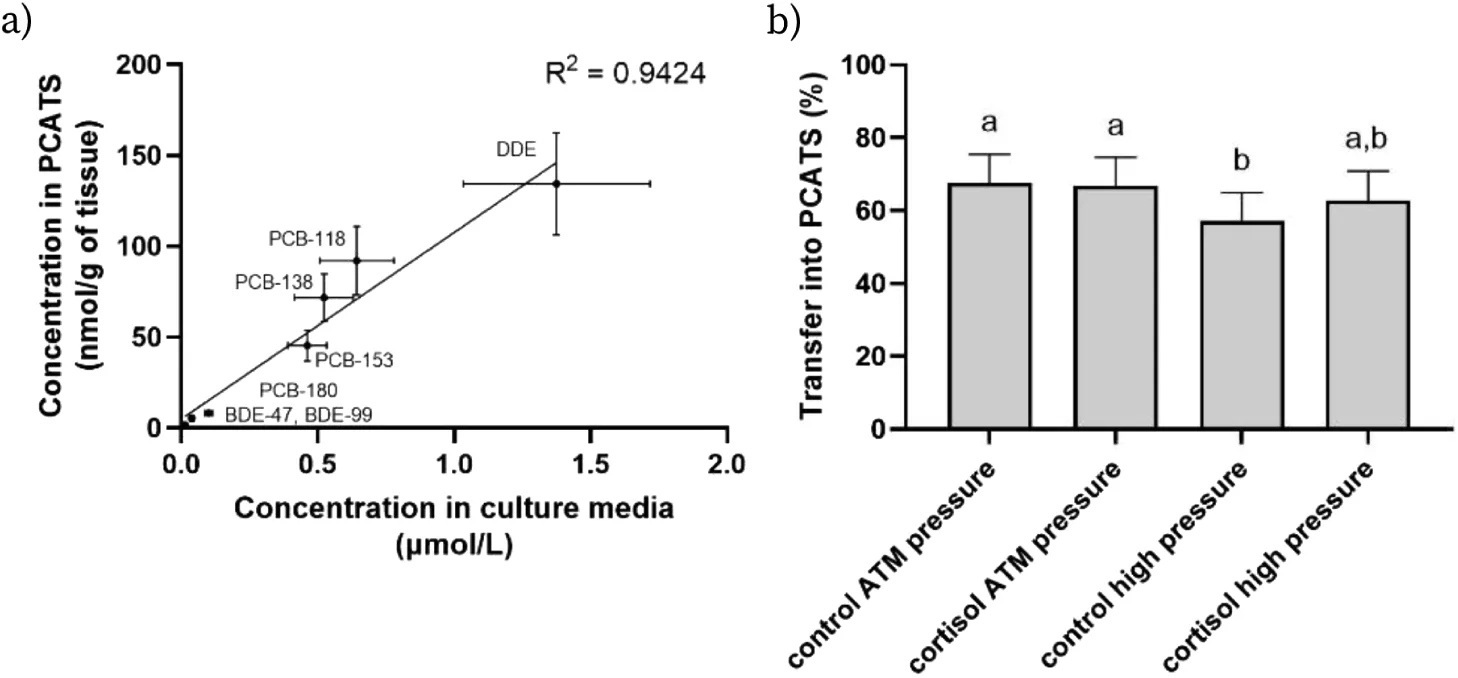
Transfer of persistent organic pollutants or POPs (4,4’-DDE, Aroclor 1254 mainly composed of PCBs as well as BDE-47 and BDE-99) into precision-cut adipose tissue slices (PCATS) of northern elephant seals. (a) Correlation between concentration of POPs in culture media and PCATS tissue collected after POP treatment for 45 h (coefficient of determination *R* ^2^ = 0.94). Each dot represents mean ± SEM concentration of each POP in PCATS obtained from six early weaned male pups and their culture media. (b) Impact of high-pressure cycles and cortisol treatment on the transfer of POPs from culture medium into PCATS (%). Mean + SEM from PCATS from six early weaned male pups are shown. PCATS were exposed to high-pressure cycles either alone or in combination with 2 μM cortisol. Controls at atmospheric pressure (ATM) with and without cortisol were run in parallel. Significant differences between treatments are represented by different lowercase letters (adj. *p* < 0.05).

### 3.3 Stress Impact on Glycerol Release and Fatty Acid Profiles in PCATS

Stress treatments significantly influenced glycerol release (F_3,15_ = 36.00, *p* < 0.0001) (Figure 6). Specifically, epinephrine alone or combined with chronic stress treatment induced higher glycerol release than control or chronic stress treatment alone (all comparisons: *p* < 0.0001).

**Figure 6:**
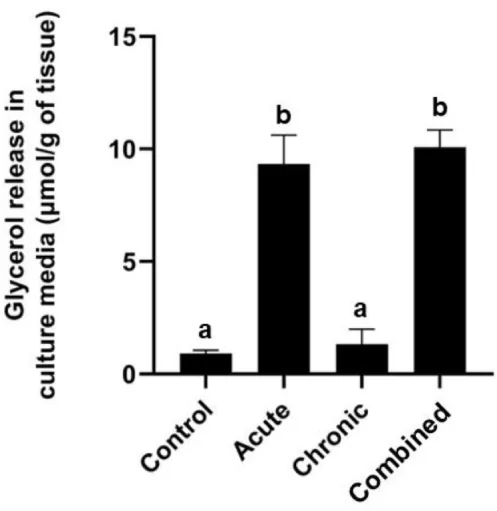
Impact of stress treatments on glycerol release from precision-cut adipose tissue slices (PCATS) of northern elephant seals (*n* = 6 male pups). PCATS were exposed to (i) acute stress (epinephrine applied after 36 h), (ii) chronic stress (45 h-long exposure to cortisol and a mixture of persistent organic pollutants combined with 6 high-pressure cycles up to 30 bar lasting 20 min each after 36 h), (iii) combined stress treatment (acute combined to chronic stressors) and (iv) control (standard culture). Data are mean + SEM for PCATS from six early weaned male pups. Triplicates were run for each pup. Different lowercase letters highlight significant differences between treatments (adj. *p* < 0.0001).

Fatty acids C14:0, 16:0, 16:1 n-7, 18:0, 18:1 n-7, 18:1 n-9 (cis), 18:2 n-6, 20:1 n-9, and C22:6 n-3 accounted for more than 90% of all fatty acids present in neutral lipid and phospholipid fractions (Table S4). Exposure to acute, chronic or combined stress treatments did not affect fatty acid profiles in PCATS (*p* > 0.05).

### 3.4 Stress Impact on Leptin Secretion by PCATS

We evaluated the impact of stress treatments on the secretion of leptin, a major regulatory adipokine involved in energy homeostasis (Figure 7). Experimental treatments significantly impacted leptin release in the culture media (F_3,12_ = 6.15, p = 0.0089). PCATS exposed to acute stress treatment alone secreted less leptin than controls (p = 0.043) or those exposed to chronic stress treatment (p = 0.014).

**Figure 7:**
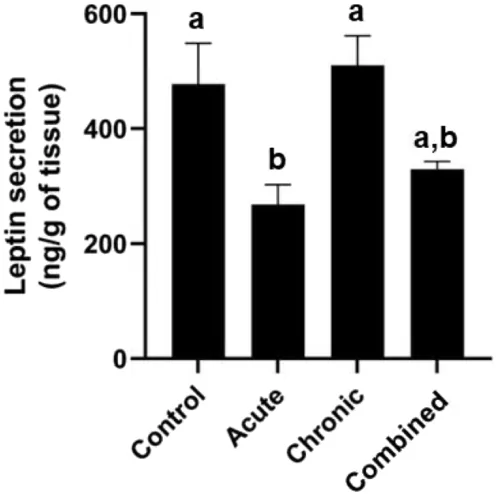
Impact of stress treatments on leptin secretion from precision-cut adipose tissue slices (PCATS) of northern elephant seals (*n* = 5 male pups). PCATS were exposed to (i) acute stress (epinephrine applied after 36 h), (ii) chronic stress (45 h-long exposure to cortisol and a mixture of persistent organic pollutants combined with 6 high-pressure cycles up to 30 bar lasting 20 min each after 36 h), (iii) combined stress treatment (acute combined to chronic stressors) and (iv) control (standard culture). Data are mean + SEM for PCATS from five early weaned male pups. Different lowercase letters show significant differences between treatments (adj. *p* < 0.05).

## 4 Discussion

This study presents a novel approach to characterize the toxicological impacts of human activities on marine mammal blubber transcriptome. By overcoming the practical and ethical limitations of *in vivo* studies, exposure of NES PCATS to stress-response hormones and POPs, under simulated deep-diving hydrostatic pressure, provides an efficient and realistic way to assess the combined impacts of multiple stressors.

Our *ex vivo* observations align with *in vivo* studies in NES, showing that serum POP concentrations are predictive of blubber concentrations.^52^ Yet, these results should be taken cautiously as serum contaminant concentrations vary with the animal’s ecology (e.g., fasting, reproductive status) and are more dynamic than those measured in adipose tissue.^53,54^ High-pressure cycles significantly decreased the incorporation of pollutants into the tissues, likely due to the negative impact of pressure on membrane fluidity.^26^ Retention of POPs in the blood circulation could alter their distribution among tissues. However, physiological implications remain unclear. Further research should determine whether transient changes in POP uptake by blubber during diving could influence contaminant distribution during feeding at sea. These hypotheses should be taken cautiously given the relatively small magnitude of the decrease in POP incorporation observed here and limited sample size.

At the transcriptomic level, the downregulation of genes associated with xenobiotic metabolism in response to acute stress (epinephrine exposure) was in line with previous studies of rodent and human hepatocytes exposed to catecholamines.^55^ In primary human hepatocytes exposed to 10 μM epinephrine for 24 h, expression of drug transporters was repressed.^56^ Similarly, primary rat hepatocytes exposed to 10 μM epinephrine for 24 h showed a 5-fold downregulation of cytochrome P450 (*CYP2C11*), a key detoxification enzyme primarily expressed in the liver.^57,58^ This regulation may involve β2-adrenergic receptor (β2-AR)-mediated induction of pro-inflammatory cytokine *IL6*, which is known to downregulate cytochrome P450 expression.^57,59^ In the present study, *IL6* was upregulated and *CYP1A1* was downregulated in PCATS exposed to epinephrine alone, supporting the proposed β2-AR/IL6 regulatory pathway. However, to our knowledge, there is no study comparing detoxification processes between adipose tissue and liver in marine mammals. Evidence from human subcutaneous adipose tissue explants and primary culture of hepatocytes exposed to P450 inducers showed differential regulation of cytochrome P450 expression (family 1) and aryl hydrocarbon receptor in both tissues, suggesting that detoxifying mechanisms occurring in liver are also functional in adipose tissue, but to a considerably lower degree.^60^ Thus, changes in xenobiotic metabolism-related genes in PCATS may predict similar responses in the liver during acute stress. The present findings suggest weaker detoxification capacities during acute stress, raising health concerns for highly contaminated populations.

In contrast with acute stress, chronic stress treatment upregulated genes associated with xenobiotic metabolism in PCATS. Even though no previous studies investigated the combined impacts of a mixture of POPs, cortisol and variable hydrostatic pressure *ex vivo*, our findings are consistent with studies on their individual effects. First, *CYP1A1* upregulation may result from dioxin-like PCBs in the Aroclor 1254 mixture, which activate Aryl hydrocarbon receptor (AhR). Many correlative studies in marine mammals highlighted positive relationships between pollutant concentrations and the expression of genes related to xenobiotic metabolism. Indeed, CYP induction is widely used as a biomarker of contaminant exposure in marine mammals.^61^ For example, highly contaminated populations of ringed seals (*Phoca hispida*) and gray seals (*Halichoerus grypus*) exhibited greater CYP1A induction than less contaminated populations.^62^ Similarly, *CYP1A1* expression in liver from stranded cetacean was also correlated with pollutant levels in blubber.^63^ *AhR* expression in northern fur seal (*Callorhinus ursinus*) blubber was positively associated with PCBs, PBDEs, and HCB concentrations while *CYP1A* was positively correlated with PCB levels only.^64^ The other stressors (i.e., cortisol and hydrostatic pressure) in our treatment may also have contributed to *CYP1A* upregulation. For instance, the expression of genes encoding *CYP* enzymes (*CYP2* and *CYP3* family members) was upregulated in rat primary hepatocytes exposed to 25 μM corticosterone as well as in hepatocyte cell lines (HepG2) exposed to hydrocortisone.^65,66^ Regarding hydrostatic pressure, our results align with a study on human hepatocytes, showing an upregulation of *CYP1A2* in cells exposed to hydrostatic pressure.^67^ Conversely, in precision-cut liver slices from the deep-sea fish *Coryphaenoides rupestris* exposed to an AhR agonist, *CYP1A* was upregulated at atmospheric pressure and downregulated at high hydrostatic pressure (5–15 MPa), highlighting the relevance of investigating the effects of pressure on xenobiotic metabolism.^39^ In the present study, individual stressor effects could not be assessed as chronic stressors were applied in combination. Future studies are needed to disentangle the individual effects of POPs, cortisol and variable hydrostatic pressure on xenobiotic metabolism in marine mammals.

When both acute and chronic stress treatments were applied to the elephant seal PCATS, *CYP1A1* expression remained upregulated, indicating that chronic effects outweighed the inhibitory influence of epinephrine. However, the combined stress treatment did not significantly alter the xenobiotic metabolism pathway from the GSEA in PCATS, unlike the acute and chronic stress treatments applied separately. This experiment reflects complex interactions between multiple stressors and suggests an antagonistic effect of stressors on some pathways, resulting in failure to upregulate them when stressors co-occur. In marine mammals exposed to multiple stressors simultaneously, impaired biotransformation capacity (potentially as a consequence of low expression of genes in this pathway) could induce serious health implications, especially in highly contaminated species such as killer whales (*Orcinus orca*)^68^ and deep-diving species such as sperm whales (*Physeter macrocephalus*) and long-finned pilot whales (*Globicephala melas*).^69^

The stimulating effect of epinephrine, applied alone or combined with chronic stressors, on the release of glycerol by NES PCATS is consistent with previous findings, highlighting the key role of catecholamines in lipolysis induction in NES blubber.^10,31^ The binding of catecholamines to adrenergic receptors (ADRB2, ADRB3), located on the plasma membrane of adipocytes, triggers a cascade of reactions resulting in the breakdown of triglycerides in a three-step process catalyzed by adipose triglyceride lipase (ATGL), hormone sensitive lipase (HSL) and monoglyceride lipase (MGL) to produce glycerol and free fatty.^12,70^ In addition to the post-translational effect of epinephrine on lipolysis induction, the transcriptomic analysis of epinephrine-exposed PCATS revealed a lack of sensitivity of lipolysis enzymes to epinephrine. While adrenergic receptors (*ADRB2*, *ADRB3*) were upregulated, the enzyme *MGL* was downregulated and *ATGL* and *HSL* expression remained unchanged in PCATS treated with epinephrine. In fasting NES adult females and weaned pups, ATGL protein levels increased while HSL abundance did not change in blubber over the duration of the fast,^46^ suggesting that these transcriptional patterns may reflect adaptative mechanisms for energy management to prolonged periods of food deprivation in fasting-adapted species. Post-translational adaptations were also observed in lactating NES (e.g., chaperone and inhibiting proteins).^71^ The catabolic effect of epinephrine was further supported by downregulation of adipogenesis-related genes including *PPARG*, a key transcription factor, in PCATS exposed to epinephrine alone or combined with chronic stressors. Similarly, genes involved in lipid synthesis, desaturation and elongation were downregulated under acute and combined stress conditions, supporting the shift toward catabolism and potentially explaining the lack of modifications in fatty acid profiles in exposed PCATS. The impact of epinephrine on lipogenesis in marine mammals remains poorly documented. Interestingly, a previous study on NES PCATS reported upregulation of PPARγ coactivators (*PPARGC1A* and *LPIN1*) and downregulation of its corepressor *ASXL1* following epinephrine exposure but no regulation of *PPARG* was found.^23^ Differences in experimental setups between the present work and this previous study should be highlighted as culture duration was slightly longer (48 h), the number of individuals was lower (n = 3) and only females were used in that study while males were sampled for the present work. Yet, the present result aligns with studies conducted on human, rodent and 3T3-L1 adipocyte cell lines showing that catecholamines suppress fatty acid re-esterification and *PPARG* expression.^72,73^ The similar transcriptional responses observed under acute and combined stressors suggest that epinephrine is the main driver of changes in expression of these lipid metabolism-related genes.

In contrast to acute and combined stressors, chronic stressors did not affect expression of lipolysis- or lipogenesis-associated genes in NES PCATS. Yet, *ADRB2* was upregulated, suggesting that stressors may partly influence energy metabolism at the transcriptomic level. As cortisol, POPs and variable hydrostatic pressure were not investigated separately, their specific contributions to energy homeostasis could not be disentangled. Nevertheless, our findings align with previous studies investigating the impact of glucocorticoids on lipid metabolism. For instance, genes related to lipolysis (e.g., *ADRB2*) were upregulated in NES blubber following ACTH injection and subsequent increase in circulating cortisol 2 h postinjection.^30^ In that study, adipogenesis-related genes (e.g., *PPARG*) were also upregulated, which was not the case in the present work, reflecting differences between *in vivo* and *ex vivo* approaches, durations of exposure and the treatments applied which differed between studies. Regarding POPs, most studies focus on adipo- and lipogenesis.^74–77^ DDE, the most abundant component of the mixture applied to NES PCATS, as well as BDE-47 and −99 have been shown to induce adipo- and lipogenesis in adipose tissue,^78,79^ similar to cortisol.^80^ Conversely, Aroclor 1254, also present in our mixture, downregulated *PPARG* and lipid accumulation in human adipose mesenchymal stem cells exposed to 10 μM.^81^ To our knowledge, no study has ever investigated the impact of variable hydrostatic pressure on lipid metabolism in marine mammals. The lack of changes in expression of genes involved in adipo- and lipogenesis in POP-treated NES PCATS could result from antagonistic effects of the different chronic stressors on this biological process, reflecting complex interactions between them. Further studies applying chronic stressors individually are needed to confirm this hypothesis.

The inhibitory effect of epinephrine on leptin secretion is consistent with previous results on NES PCATS^10,23,31^ as well as *in vivo* and *in vitro* studies conducted in biomedical models.^82–85^ Transcriptomic data indicate that this effect originates at the *LEP* gene level, likely through a cAMP-dependent transcription factor (i.e., CREB) mechanism.^86^ Conversely, chronic stressors had no impact on leptin secretion, despite an upregulation of LEP transcription, which aligns with (i) studies of pinnipeds exposed to exogenous administration of ACTH and subsequent increase in cortisol levels, which upregulated *LEP* ^87,88^ and (ii) positive correlation between blubber cortisol and *LEP* expression in humpback whales (*Megaptera novaeangliae*).^89^ The present results also corroborate with studies conducted on 3T3-L1 adipocytes exposed to PCBs or 4,4’-DDE showing an upregulation of *LEP* expression,^32,33,90,91^ as well as correlative studies between circulating PBDEs and *LEP* expression in human subcutaneous adipose tissue.^92^ Even though epinephrine likely drove the downregulation of *LEP* expression in combined stress-exposed PCATS, the secretion of the adipokine protein remained unchanged, echoing previous findings showing the antagonistic effects of cortisol and epinephrine on leptin in seal blubber.^31^ The present findings suggest that chronic stressors may modulate epinephrine-mediated leptin regulation. Such dysregulation of leptin production by multiple combined stressors could alter energy homeostasis in marine mammals, which encounter frequent periods of fasting, associated with elevated circulating levels of cortisol and POPs.^93,94^

The downregulation of *ADIPOQ* by epinephrine is surprising, given its known stimulatory effect on adiponectin secretion through adrenergic stimulation.^95^ Measurements of secreted adiponectin are needed to determine whether this change in gene expression is mirrored at the protein level. Chronic stressors had no effect on *ADIPOQ* expression, although cortisol alone has been previously shown to upregulate this gene in NES PCATS.^31^ Similarly, a correlative study in humpback whales showed that elevated levels of blubber cortisol were positively related to *ADIPOQ*.^89^ POPs have had varying effects on *ADIPOQ* expression in rodent and human cells: *ADIPOQ* was downregulated by PCB-77 and Aroclor 1254^82,97^, unchanged by PCB-153^97^ and upregulated by BDE-99 and 4,4’-DDE.^97,98^ The lack of change in *ADIPOQ* expression in NES PCATS response to the combined stressors may reflect their antagonistic effects.

All stress treatments upregulated inflammation-related genes, confirming stressor impact on this process. Epinephrine stimulated expression of *IL6* and genes associated with the pathway “TNFα signaling via NFκB”, consistent with studies in rodents and humans.^99–103^ Chronic stressors also increased expression of some inflammation-related genes in NES PCATS but had no impact on the expression of interleukins, possibly due to cortisol’s immunosuppressive role.^89,104^ POPs, however, have been shown to have a pro-inflammatory effect on human and rodent adipose tissue by increasing macrophage infiltration^105^ and upregulating interleukin expression *in vitro*.^32,96,106,107^ In marine mammals, expression of a pro-inflammatory cytokine, *IL-2*, has been correlated with POP contamination, including PCBs, in crabeater seals (*Lobodon carcinophaga*) and harbor seals (*Phoca vitulina*).^108,109^ Overall, chronic stressor-driven inflammation appears POP-mediated, which may heighten susceptibility to inflammatory disease in exposed populations of marine mammals.^110,111^ Combined stressor effects on gene expression are likely the result of interactions between epinephrine and POPs, highlighting the complexity of stressor interplay in marine mammals.

Genes related to hypoxia were upregulated while those associated with peroxisomes, the oxidative organelles involved in reactive oxygen species (ROS) metabolism,^112^ were downregulated in PCATS in response to all stress treatments. Hypoxia is known to alter lipid metabolism, reduce lipolytic signaling, and promote inflammation and tissue damage in humans.^113,114^ Marine mammals, however, have developed physiological adaptations to cope with hypoxia, such as cardiovascular control, efficient oxygen transport and storage and high antioxidant capacity.^114,115^ In cells from terrestrial mammals, acute hypoxia does not impact the abundance of peroxisomes, but prolonged hypoxia significantly reduces their number.^112^ The upregulation of hypoxia-related genes by epinephrine aligns with its vasoconstricting effect *in vivo* ^116^ though the mechanism by which epinephrine upregulates hypoxia-related genes in adipocytes of a deep-diving mammal remains unclear. Importantly, a study on human endothelial cells reported that >90% of hypoxia-regulated genes were also altered by epinephrine,^117^ suggesting shared targets rather than a cellular response to hypoxia. Chronic stressors also upregulated hypoxia-related genes in NES PCATS, possibly through overlapping signaling pathways. In biomedical models, (i) POPs and hypoxia both stimulate the aryl hydrocarbon receptor nuclear translocator (ARNT) transcription factor, recruited by AhR and hypoxia inducible factor 1α (HIF-1α)^118–120^ and (ii) glucocorticoids have also been shown to interact with hypoxia signaling pathways.^121^ Whether these changes reflect actual hypoxia or transcriptional crosstalk remains unresolved. Data on hydrostatic pressure effects on expression of similar genes are lacking. Further experiments, such as measuring markers of hypoxia in PCATS, are needed to better characterize the impact of multiple stressors on this process in blubber.

This study had several limitations, including a sample size constrained by laboratory and field limitations. Nevertheless, the number of NES pups sampled was sufficient to detect changes in POP uptake, metabolism, gene expression, and protein secretion in stress-exposed PCATS. Blubber availability per pup limited the range of experimental conditions. Future studies should aim to disentangle the individual effects of chronic stress components and extend exposure to variable-pressure to better mimic natural diving patterns. Although the POP mixture used here represented the major compounds found in NES blubber, blubber contains a complex cocktail of contaminants whose concentrations vary with life history, diet, location, sex, age and physiological states.^93,122^ Recent findings of highly fluorinated per- and polyfluoroalkyl substances in killer whale blubber,^123^ highlight the need for future studies using complete contaminant extract from NES blubber for more realistic exposure scenarios.^124,125^ Because POP transfer from culture media into PCATS was high, lower exposure concentrations may better reflect levels found in blubber. However, as exposure is conducted over short periods compared with the chronic exposure experienced by free-ranging animals, somewhat elevated concentrations may remain relevant for investigating contaminant effects within the temporal constraints of the model. Finally, comparing PCATS responses across populations, age classes, and sexes, as well as examining POP transfer dynamics and the influence of exposure duration on transcriptomic profiles, would further improve our understanding of stressor effects on marine mammals.

The synergistic and antagonistic interactions of treatments on gene expression observed in this study underline the complex and, still, poorly understood ways in which stressors interplay. These findings suggest that anthropogenic stressors may alter blubber functionality at multiple levels. They also provide new insights into the biomolecular effects of anthropogenic stressors on marine mammal physiology and raise concerns about increased POP toxicity under physiological stress. More broadly, this paper also underscores the need for integrative studies addressing multiple threats, including infectious diseases and emerging contaminants, to better understand their impacts on marine mammal physiology at both tissue and organismal levels.

## Supplementary Material



## Data Availability

Supplementary files related to *de novo* transcriptome assembly (assembly, blast and gene count information), differential expression analysis (DEA) and enrichment analysis (GSEA) are available at https://doi.org/10.14428/DVN/TOYCCT.

## Notes

Supporting Information (word document) contains sample information, a summary of POP quantification across different marine mammal species, pollutant mixture composition and hyperbaric system setup, as well as pollutant analysis, analysis of lipid fractions and RNA isolation and sequencing methods. (PDF)
