# Supplementary material for: Elephant Seal Blubber Under Multiple Stressors: Persistent Organic Pollutants, Stress Hormones and Hydrostatic Pressure Alter Transcriptome, Lipolysis and Leptin Secretion
Source: Environ Sci Technol. 2026 Aug 31;60(37):26131–44. doi: 10.1021/acs.est.6c05765 (PMC13614386; doi:10.1021/acs.est.6c05765)
Supplement: Supplementary file 1 [file es6c05765_si_001.pdf]

SUPPORTING INFORMATION

# Elephant seal blubber under multiple stressors: persistent organic pollutants, stress hormones and hydrostatic pressure alter transcriptome, lipolysis and leptin secretion

*Laura Pirard[a], Jane I. Khudyakov[b], Manon Martin[a], Louise Vanoost[a], Aurélien Warnant[a], Delphine Decloux[a], Thomas Baily[a], Daniel E. Crocker[c], Gauthier Eppe[d], Georges Scholl[d], Melissa M. Page[a], Jean-François Rees[a], Donald R. Smith[e] and Cathy Debier[a]**

[a]Louvain Institute of Biomolecular Science and Technology, UCLouvain, Louvain-la-Neuve, 1348, Belgium
[b]Department of Biological Sciences, University of the Pacific, Stockton, CA, 95211, United States
[c]Department of Biology, Sonoma State University, Rohnert Park, CA, 94928, United States
[d]Mass Spectrometry Laboratory, CART, MolSys Research Unit, University of Liège, Liège, 4000, Belgium
[e]Microbiology and Environmental Toxicology Department, University of California Santa Cruz, Santa Cruz, CA, 95064, United States

*Corresponding author
Email: cathy.debier@uclouvain.be

Number of pages: 17

Number of figures: 1

Number of tables: 4

**Table S1.** Early weaned male northern elephant seal pups sampled for precision-cut adipose tissue slice (PCATS) experiments and downstream analyses.

| PUP ID | Analyses |
|---|---|
| 1 | POP quantification, glycerol measurement, leptin measurement, transcriptomic analysis and viability assay |
| 2 | POP quantification, glycerol measurement, leptin measurement and transcriptomic analysis |
| 3 | Transcriptomic analysis |
| 4 | Transcriptomic analysis |
| 5 | POP quantification |
| 6 | Glycerol measurement, leptin measurement and transcriptomic analysis |
| 7 | POP quantification, glycerol measurement, leptin measurement and lipid content |
| 8 | POP quantification, glycerol measurement, leptin measurement and lipid content |
| 9 | POP quantification, glycerol measurement and lipid content |

**Table S2.** Concentrations of persistent organic pollutants (PCBs, p,p′-DDE and PBDEs) measured in serum (if available) and blubber from marine mammals.

| Pollutant | Marine mammal species | Measured concentrations (ng/g, lw) | Location and reference |
|---|---|---|---|
| | | Serum (range) | |
| ∑PCBs | Northern elephant seal (*Mirounga angustirostris*) | Pups - early fast (mean ± SEM): 372 ± 17<br>Pups – late fast (mean ± SEM): 585 ± 35 | California, USA [1] |
| | Northern elephant seal (*Mirounga angustirostris*) | Early breeding adults (Mean [range]): 838 [511–1495]<br>Late breeding adults (Mean [range]): 818 [542–1362] | California, USA[2] |
| | | Adipose tissue (range) | |
| ∑PCBs | Grey seal (*Halichoerus grypus*) | Adult females - early lactation inner (Mean [range]): 1260 [580 – 2820)]<br>Adult females – late lactation inner (Mean [range]): 3240 [1160 – 10090] | UK[3] |
| | Grey seal (*Halichoerus grypus*) | Pups – full biopsy (Mean [range]): 432 [219.9 – 2192.9] | UK[4] |
| | Polar bear (*Ursus maritimus*) | Adult females (Mean [range]): 4111 [2000 – 8453]<br>Adult males (Mean [range]): 4276 [3236 – 5649] | Canada[5] |
| | Polar bear (*Ursus maritimus*) | Adults (Mean [confidence interval]): 5523 [4617–6608] | Canada[6] |
| | Ringed seal (*Pusa hispida*) | Adults (Mean [confidence interval]): 338.1 [287.7–397.2] | Canada[6] |
| | Harbor seals (*Phoca vitulina*) | Adult females – full biopsy (Median [range]): 497 [355–800]<br>Adult males – full biopsy (Median [range]): 474 [274–658]<br>Juveniles – full biopsy (Median [range]): 274 [199–286] | Svalbard, Norway[7] |
| | Killer whale (*Orcinus orca*) | Adult females (mean ± SD): 1200 ± 910<br>Adult males (mean ± SD): 18000 ± 15000 | Russia[8] |
| | Killer whale (*Orcinus orca*) | Adults (Mean [range]): 1060 (420 – 3970) | Antarctica[9] |
| | Killer whale (*Orcinus orca*) | Adult males (mean [Confidence interval]): 26940 [16613 – 43687] | Norway[10] |
| | Sperm whale | One individual: 1970 | Germany[11] |

| Pollutant | Marine mammal species | Measured concentrations (ng/g, lw) | Location and reference |
|---|---|---|---|
| | (*Physeter macrocephalus*) | | |
| | Southern Elephant seal (*Mirounga leonina*) | Adult females - outer blubber (mean): 29.5<br>Pups – outer blubber (mean): 16.09 | Antarctica[12] |
| | Northern Elephant seal (*Mirounga angustirostris*) | Early breeding adults (Mean [range]):<br>Inner blubber: 690 [473–1120]<br>Outer blubber: 900 [504–1417]<br><br>Late breeding adults (Mean [range]):<br>Inner blubber: 1241 [495–2502]<br>Outer blubber: 912 [497–1461] | California, USA[2] |
| | Northern Elephant seal (*Mirounga angustirostris*) | Early fast pups: (mean ± SEM)<br>Inner blubber: 436 ± 25<br>Outer blubber: 419 ± 27<br><br>Late fast pups: (mean ± SEM)<br>Inner blubber: 1060 ± 71<br>Outer blubber: 732 ± 45 | California, USA[1] |
| PCB-153 | Grey seal (*Halichoerus grypus*) | Pups - full-biopsy (median [range]): 226.3 [110.7–1108.9] | UK[4] |
| | Polar bear (*Ursus maritimus*) | Adult females (Mean [range]): 1489 [771 – 2951]<br>Adult males (Mean [range]): 1581 [1225 – 2042] | Canada[5] |
| | Polar bear (*Ursus maritimus*) | Adults (Mean [confidence interval]): 1937 [1927–1948] | Canada[6] |
| | Ringed seal (*Pusa hispida*) | Mean [confidence interval]: 43.0 [33.4–55.3] | Canada[6] |
| | Killer whale (*Orcinus orca*) | Adult males (mean [Confidence interval]): 6766 [4020-11388] | Norway[10] |
| | Southern Elephant seal (*Mirounga leonina*) | Adult female – outer blubber (mean ± SEM): 9.33 ± 1.55<br>Pup – outer blubber (mean ± SEM): 4.80 ± 0.90 | Antarctica[12] |
| PCB-118 | Southern Elephant seal (*Mirounga leonina*) | Adult female – outer blubber (mean ± SEM): 2.92 ± 1.27<br>Pup – outer blubber (mean ± SEM): 2.21 ± 0.85 | Antarctica[12] |

| Pollutant | Marine mammal species | Measured concentrations (ng/g, lw) | Location and reference |
|---|---|---|---|
| | Grey seal (*Halichoerus grypus*) | Pups – full biopsy (median [range]): 9.8 [4.1– 63.1] | UK[4] |
| | Killer whale (*Orcinus orca*) | Adult males (mean [Confidence interval]): 1430 [1062-1926] | Norway[10] |
| PCB-138 | Grey seal (Halichoerus grypus) | Pups – full biopsy (median [range]): 128.1 [66.8–526.9] | UK[4] |
| | Killer whale (*Orcinus orca*) | Adult males (mean [Confidence interval]): 3 [2.4-3.7] | Norway[10] |
| | Southern Elephant seal (*Mirounga leonina*) | Adult female – outer blubber (mean ± SEM): 6.39 ± 1.07<br>Pup – outer blubber (mean ± SEM): 3.44 ± 0.65 | Antarctica[12] |
| PCB-180 | Killer whale (*Orcinus orca*) | Adult males (mean [Confidence interval]): 2358 [1409-3943] | Norway[10] |
| | Grey seal (*Halichoerus grypus*) | Pups – full biopsy (median [range]): 35.1 (16.5 – 479.9) | UK[4] |
| | Southern Elephant seal (*Mirounga leonina*) | Adult female – outer blubber (mean ± SEM): 3.23 ± 0.32<br>Pup – outer blubber (mean ± SEM): 1.62 ± 0.46 | Antarctica[12] |
| 4,4′-DDE | Grey seal (*Halichoerus grypus*) | Pups – full biopsy (median [range]): 185.4 [111.5–477.9] | UK[4] |
| | Polar bear (*Ursus maritimus*) | Adult females (Mean [range]): 121 [87.7–168]<br>Adult males (Mean [range]): 131 [98.4–175] | Canada[5] |
| | Killer whale (*Orcinus orca*) | Adult males (Mean ± SEM): 30000 (16000 – 49000) | North Pacific – Alaska, USA[13] |
| | Killer whale (*Orcinus orca*) | Adult males (mean [Confidence interval]): 11652 [6966-19490] | Norway[10] |
| | Polar bear (*Ursus maritimus*) | Adults (Mean [confidence interval]): 113 [112–114] | Canada[6] |
| | Ringed seal (*Pusa hispida*) | Adults (Mean [confidence interval]): 128.8 [105.9–157.0] | Canada[6] |
| | Northern Elephant seal (*Mirounga angustirostris*) | Early fast pups (mean ± SEM):<br>Inner blubber: 946 ± 66 | California, USA[1] |

| Pollutant | Marine mammal species | Measured concentrations (ng/g, lw) | Location and reference |
|---|---|---|---|
| | | Outer blubber: 928 ± 62<br><br>Late fast pups (mean ± SEM):<br>Inner blubber: 1658 ± 145<br>Outer blubber: 1147 ± 84 | |
| | Southern Elephant seal (*Mirounga leonina*) | Adult female – outer blubber (mean ± SEM): 108.5 ± 19.5<br>Pup – outer blubber (mean ± SEM): 78.6 ± 13.7 | Antarctica[12] |
| ΣPBDE | Killer whale (*Orcinus orca*) | Female (mean ± SD): 0.00<br>Male (mean ± SD): 13 ± 17 | Russia[8] |
| | Killer whale (*Orcinus orca*) | Mean (range): 39.5 [18.4–138] | Antarctica [9] |
| | Killer whale (*Orcinus orca*) | Adult males (mean [Confidence interval]): 475 [348 - 647] | Norway[10] |
| | Northern Elephant seal (*Mirounga angustirostris*) | Early fast pups (mean ± SEM):<br>Inner blubber: 10 ± 1<br>Outer blubber: 11 ± 1<br><br>Late fast pups (mean ± SEM):<br>Inner blubber: 19 ± 3<br>Outer blubber: 13 ± 2 | California, USA[1] |
| | Ringed seal (*Pusa hispida*) | Adults Mean [confidence interval]: 10.0 [8.4–11.9] | Canada[6] |
| | Grey seal (*Halichoerus grypus*) | Pups – full biopsy (median [range]): 16.4 [7.6–69.2] | UK[4] |
| | Polar bear (*Ursus maritimus*) | Adults (Mean [confidence interval]): 78.4 [65.6–93.6] | Canada[6] |
| | Polar bear (*Ursus maritimus*) | Adult females (Mean [range]): 88.5 [34.5–227]<br>Adult males (Mean [range]): 55.5 [39.6–77.6] | California, USA[1] |
| BDE-47 | Northern Elephant seal (*Mirounga angustirostris*) | Early fast pups (mean ± SEM):<br>Inner blubber: 8.5 ± 1.0<br>Outer blubber: 8.4 ± 1.2 | California, USA[1] |

| Pollutant | Marine mammal species | Measured concentrations (ng/g, lw) | Location and reference |
|---|---|---|---|
| | | Late fast pups (mean ± SEM):<br>Inner blubber: 15.4 ± 2.2<br>Outer blubber: 10.4 ± 1.3 | |
| | Grey seal<br>(*Halichoerus grypus*) | Pups – full biopsy (median [range]): 13.5 [6.2–60.9] | UK[4] |
| | Killer whale<br>(*Orcinus orca*) | Adult males (mean [Confidence interval]): 275 [204 – 370] | Norway[10] |
| | Polar bear<br>(*Ursus maritimus*) | Adult females (Mean [range]): 31.7 [13.3–75.5]<br>Adult males (Mean [range]): 22.1 [15.2–32.3] | Canada[5] |
| BDE-99 | Grey seal<br>(*Halichoerus grypus*) | Pups – full biopsy (median [range]): 0.4 [0.1–4.1] | UK[4] |
| | Killer whale<br>(*Orcinus orca*) | Adult males (mean [Confidence interval]): 76.1 [53.8 – 107.6] | Norway[10] |
| | Northern Elephant seal<br>(*Mirounga angustirostris*) | Early fast pups (mean ± SEM):<br>Inner blubber: 0.4 ± 0.1<br>Outer blubber: 0.6 ± 0.1<br><br>Late fast pups (mean ± SEM):<br>Inner blubber: 1.0 ± 0.1<br>Outer blubber: 0.6 ± 0.1 | California, USA[1] |

**Table S3.** Concentration of indicator PCBs in Aroclor 1254 (DRE-C20125400, lot #1120968, Dr. Ehrenstorfer, LGC Group, Augsburg, Germany)

| **IUPAC no.** | **Structure** | **No. of Cl[a]** | **Mass concentration (mg/g)** |
|---|---|---|---|
| 28 | 2,4,4´ | 3 | 2.38 |
| 52 | 2,2´,5,5´ | 4 | 55.03 |
| 101 | 2,2´,4,5,5´ | 5 | 68.09 |
| 118 | 2,3´,4,4´,5 | 5 | 65.08 |
| 138 | 2,2´,3,4,4´,5´ | 6 | 44.80 |
| 153 | 2,2´,4,4´,5,5´ | 6 | 33.59 |
| 180 | 2,2´,3,4,4´,5,5´ | 7 | 7.20 |

[a] Number of chlorine substitutions.

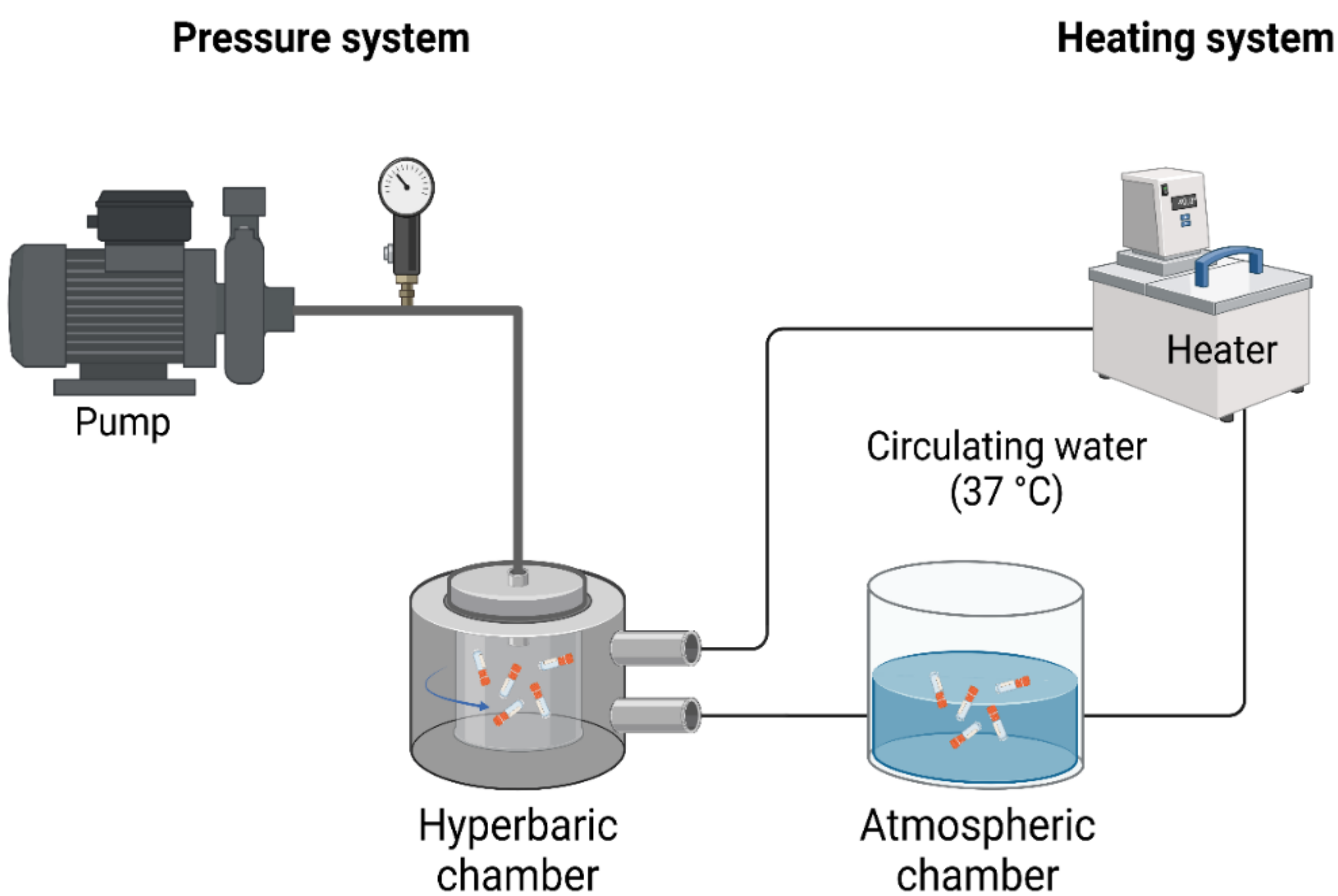

**Figure S1.** Illustration of the hyperbaric system used for culture of precision-cut adipose tissue slices (PCATS) from northern elephant seals. The hyperbaric system was composed of a hyperbaric chamber connected to a water pump and a manometer, an atmospheric chamber as a control, and a water heater for maintaining the temperature of both chambers at 37 °C. The hyperbaric chamber was composed of a central area where cryotubes containing PCATS were placed, surrounded by a cylindrical cavity in which warmed water circulated (blue arrow). Created in BioRender. Debier, C. (2026) https://BioRender.com/78007bh.

**Analysis of POPs transferred from culture media into precision-cut adipose tissue slices (PCATS)**

To measure POPs in culture media, serum from culture media was first degraded with 99% formic acid (1:1 ratio of acid to media, v:v) for 5 minutes, followed by dilution with 1 mL water. To measure POPs in PCATS, the PCATS were first microwaved for 1 minute and lipids were solubilized in 5 mL of hexane[14], followed by sonication for 10 minutes. Both culture media and PCATS samples were diluted 10 times and underwent a cleaning step with 35 mL of hexane in a silica-alumina column. To remove traces of water in the samples, an additional cleaning step in Na2SO4 columns was performed with hexane as an eluant. Samples were then evaporated under a nitrogen flux at 30°C and resolubilized in 90 µL of n-nonane. Analyses were performed on an Agilent 6890 gas chromatograph (Palo Alto, CA, United States), coupled to an Autospec Ultima mass spectrometer (Waters, Manchester). Two µL of sample were injected at 275°C in splitless mode on an HT8 column (25 m x 0.22 mm x 0.25 µm; Trajan, Ringwood Victoria, Australia) at a 0.8 mL/minute He (6.0 purity; Air Liquide, Belgium) constant flow. The analysis was performed in Selected Ion Monitoring (SIM) mode at 10,000 mass resolution with Electronic Ionization at 35 eV. Two ions (quantifier and qualifier) were recorded for each analyte and their ion ratio was checked for interferences. The quantification of seven indicator PCBs (namely PCB-28, 52, 101,

118, 138, 153 and 180), BDE-47, BDE-99 and DDE was achieved by isotope dilution technique using 13C12-labelled analogs. Concentrations of PCBs were normalized per g of tissue wet weight. The transfer of POPs from culture media into PCATS was calculated using the following formula:

$$\text{POP transfer [\%]} = \frac{Quantity\ of\ POPs\ accumulated\ in\ PCATS\ during\ culture}{Quantity\ of\ POPs\ added\ in\ culture\ media} \times 100$$

Where "Quantity of POPs accumulated in PCATS during culture" is determined by subtracting the initial POPs quantity present in PCATS at culture start (individual initial contamination) from the final quantity present in PCATS at culture end and "$Quantity\ of\ POPs\ in\ culture\ media$" is the estimated maximal amount of POPs that could be transferred into PCATS based on its measurement in culture medium. The percent transfer was calculated for each POP individually.

**Analysis of lipid fractions in precision-cut adipose tissue slices from northern elephant seals**

Lipid content in PCATS was extracted by the Bligh and Dyer[15] method. This analysis was made on samples from three pups. One of the triplicates for each condition and each pup was randomly selected. PCATS were placed in a tube filled with 800µL of milli-Q water and silica beads and homogenized with Precellys R 24 (Bertin instruments, Montigny Le Bretonneux, France). The homogenate was mixed with 5 mL of ethanol/chloroform/water (2:2:1, v:v:v). An internal standard was added to quantify loss of triglycerides and phospholipids during extraction (i.e. heptadecanoic acid triglyceride and 1,2-dibehenoyl-sn-glycero-3-phosphocholine; Larodan, Solna, Sweden). After centrifugation, the aqueous phase was used for a second extraction with 2 mL of chloroform. The chloroform phases from the two extractions were pooled and placed on a silica column (Bond Elut, Agilent Technologies, Santa Clara, CA, United States) conditioned with hexane. Neutral lipids were eluted with chloroform/2-propanol (2:1, v/v) and phospholipids were collected in methanol. Fatty acid methyl esters (FAMEs) formed after the methylation step with KOH/MeOH 0.1% and HCl/MeOH 1,2N (5:2, v:v) were extracted with hexane/water (2:1, v:v), and an injection

standard (i.e. methyl-undecanoate; Larodan) was added to all samples. FAMEs were then separated by gas chromatography. The gas chromatograph (Trace 1310; Thermo Fisher Scientific, Milan, Italy) was equipped with an autosampler TriPlusAS (Thermo Fisher Scientific) and a Rt®-2560 capillary column (biscyanopropyl polysiloxane, 100 m length, 0.25 mm internal diameter, 0.2 µm film thickness; Restek, Bellefonte, PA, United States) with $H_2$ as carrier gas at constant pressure (200 kPa). The oven temperature program was designed as followed: 175 °C for 25 minutes, 200 °C for 20 minutes, 220 °C for 5 minutes and 235 °C for 15 minutes. The baseline temperature was 80 °C. FAMEs detection was carried out with a Flame Ionisation Detector (Thermo Fisher Scientific) maintained at 255°C and flowed by air (350 mL/minute), $N_2$ (40 mL/minute) and $H_2$ (35 mL/minute). Each peak in the chromatographs was identified and quantified by comparison to an external standard composed of 43 FAMEs (Larodan) with Chromeleon™ Chromatography Data System (CDS) Software 7.3 (Thermo Fisher Scientific). Results were expressed in µmol of fatty acids per g of tissue wet weight.

**Table S4.** Fatty acid profiles in neutral lipid and phospholipid fractions of northern elephant seal (NES) precision-cut adipose tissue slices. Mean ± SD from three NES. Precision-cut adipose tissue slices (PCATS) were exposed to multiple stressors for 45 hours in culture: i) acute stress (epinephrine applied after 36 hours), ii) chronic stress (45 hour-long exposure to cortisol and a mixture of persistent organic pollutants combined with 6 high-pressure cycles up to 30 bars lasting 20 minutes each after 36 hours), and iii) combined stress treatment (acute combined to chronic stressors). A control without stressor was run in parallel. A linear mixed model followed by Student's *t*-tests and a Šidák correction were used to compare experimental treatments. For each fatty acid, no significant difference was found between treatments ($p > 0.05$).

| Lipid fractions | Fatty acids | Control | Acute stress | Chronic stressors | Combined stressors |
|---|---|---|---|---|---|
| Neutral lipids | C10 :0 | 0.5 ± 0.0 | 0.4 ± 0.0 | 0.5 ± 0.1 | 0.3 ± 0.1 |
| (µmol/g, ww) | C12:0 | 2.7 ± 1.1 | 2.8 ± 1.3 | 3.0 ± 0.8 | 3.0 ± 2.0 |
| | C13:0 | 2.0 ± 1.7 | 1.1 ± 0.9 | 1.5 ± 1.5 | 1.0 ± 1.0 |
| | C14:0 | 57.4 ± 7.4 | 52.4 ± 10.8 | 57.0 ± 8.1 | 48.3 ± 10.1 |
| | C15:0 | 7.1 ± 0.7 | 6.5 ± 0.6 | 7.2 ± 1.0 | 5.9 ± 0.2 |
| | C16:0 | 227.9 ± 40.9 | 202.5 ± 17.2 | 225.7 ± 30.3 | 185.6 ± 19.8 |
| | C18:0 | 50.7 ± 8.1 | 39.4 ± 7.3 | 53.0 ± 8.6 | 38.5 ± 1.4 |
| | C20:0 | 0.5 ± 0.4 | 0.3 ± 0.3 | 0.5 ± 0.2 | 0.1 ± 0.1 |
| | *SFAs* | *348.7 ± 45.4* | *305.5 ± 16.5* | *348.3 ± 37.0* | *282.8 ± 19.4* |
| | C14:1n-5 | 2.3 ± 0.8 | 2.7 ± 1.3 | 2.2 ± 0.8 | 2.2 ± 0.8 |
| | C16:1 n-7 | 78.1 ± 11.6 | 87.5 ± 21.3 | 74.1 ± 7.5 | 74.9 ± 10.6 |
| | C18:1 n-7 | 54.7 ± 3.0 | 50.4 ± 6.3 | 53.8 ± 6.4 | 44.2 ± 1.8 |
| | C18:1 n-9 (cis) | 496.6 ± 140.9 | 457.1 ± 93.6 | 496.7 ± 139.8 | 413.2 ± 91.7 |
| | C18:1 n-9 (trans) | 11.7 ± 1.8 | 11.2 ± 1.9 | 11.6 ± 2.6 | 10.2 ± 1.4 |
| | C20:1 n-9 | 85.9 ± 25.8 | 75.6 ± 26.7 | 91.1 ± 35.8 | 69.4 ± 20.5 |
| | C22:1 n-7 | 4.2 ± 2.0 | 3.2 ± 1.3 | 4.4 ± 1.8 | 2.9 ± 1.3 |
| | *MUFAs* | *733.5 ± 151.7* | *687.8 ± 100.3* | *733.9 ± 172.2* | *617.1 ± 94.8* |
| | C18:4 n-3 | 2.4 ± 1.0 | 2.4 ± 1.1 | 2.5 ± 0.9 | 2.2 ± 0.9 |
| | C20:3 n-3 | 3.8 ± 0.5 | 3.6 ± 1.5 | 4.5 v 1.3 | 3.7 ± 1.7 |
| | C20:4 n-3 | 4.6 ± 1.3 | 4.3 ± 1.4 | 4.6 ± 1.2 | 3.8 ± 1.2 |
| | C20:5 n-3 | 18.8 ± 9.6 | 20.0 ± 10.6 | 18.2 ± 7.9 | 17.3 ± 8.8 |
| | C22:5 n-3 | 22.3 ± 7.5 | 20.5 ± 7.3 | 22.6 ± 6.6 | 18.8 ± 7.0 |
| | C22:6 n-3 | 78.5 ± 21.8 | 69.1 ± 18.8 | 80.5 ± 17.3 | 65.4 ± 20.7 |
| | C24:5 n-3 | 0.3 ± 0.1 | 0.3 ± 0.1 | 0.3 ± 0.1 | 0.2 ± 0.0 |

| | | | | | |
|---|---|---|---|---|---|
| | *Σω-3 PUFAs* | *130.7 ± 40.8* | *120.2 ± 38.9* | *133.1 ± 33.3* | *111.3 ± 40.2* |
| | C18:2 n-6 | 23.5 ± 2.2 | 21.7 ± 3.4 | 24.4 ± 5.1 | 19.5 ± 0.9 |
| | C18:3 n-6 | 0.1 ± 0.1 | 0.1 ± 0.1 | 0.1 ± 0.1 | 0.1 ± 0.1 |
| | C20:2 n-6 | 4.8 ± 0.3 | 4.4 ± 1.1 | 5.3 ± 1.3 | 4.1 ± 0.5 |
| | C20:3 n-6 | 1.0 ± 0.1 | 0.9 ± 0.1 | 1.1 ± 0.1 | 0.8 ± 0.1 |
| | C20:4 n-6 | 8.0 ± 0.3 | 7.7 ± 0.8 | 8.4 ± 1.1 | 7.1 ± 0.5 |
| | C22:4 n-6 | 1.9 ± 0.1 | 1.6 ± 0.1 | 2.0 ± 0.2 | 1.5 ± 0.2 |
| | C22:5 n-6 | 2.7 ± 0.2 | 2.3 ± 0.3 | 2.9 ± 0.3 | 2.2 ± 0.1 |
| | *Σω-6 PUFAs* | *42.0 ± 2.2* | *38.9 ± 5.5* | *44.1 ± 8.2* | *35.2 ± 1.0* |
| Phospholipids (nmol/g, ww) | C10 :0 | N.D. | N.D. | N.D. | 6.6 ± 11.4 |
| | C12:0 | 29.6 ± 16.5 | 41.9 ± 14.0 | 37.3 ± 41.1 | 137.7 ± 116.8 |
| | C13:0 | 18.0 ± 31.2 | N.D. | N.D. | 23.3 ± 40.3 |
| | C14:0 | 69.3 ± 13.5 | 44.2 ± 4.7 | 41.7 ± 4.3 | 32.4 ± 29.0 |
| | C15:0 | 15.3 ± 6.3 | 7.9 ± 1.2 | 11.8 ± 2.6 | 7.9 ± 7.4 |
| | C16:0 | 1272.7 ± 1126.2 | 1487.8 ± 1193.9 | 1746.3 ± 1287.6 | 1129.5 ± 910.0 |
| | C18:0 | 570.5 ± 551.3 | 709.4 ± 559.5 | 855.7 ± 617.1 | 579.8 ± 346.2 |
| | *SFAs* | *1975.4 ± 1666.8* | *2291.3 ± 1772.8* | *2692.8 ± 1930.9* | *1917.2 ± 1148.6* |
| | C16:1 n-7 | 68.5 ± 35.6 | 38.5 ± 25.3 | 16.8 ± 15.2 | 35.3 ± 31.2 |
| | C18:1 n-7 | 55.4 ± 21.5 | 32.1 ± 17.3 | 26.1 ± 9.3 | 30.4 ± 26.3 |
| | C18:1 n-9 (cis) | 502.0 ± 360.9 | 233.1 ± 128.0 | 198.3 ± 77.7 | 208.7 ± 180.7 |
| | C20:1 n-9 | 53.2 ± 34.5 | 29.5 ± 17.2 | 21.6 ± 8.8 | 29.3 ± 25.4 |
| | *MUFAs* | *679.1 ± 448.6* | *333.1 ± 186.0* | *262.9 ± 105.9* | *303.7 ± 263.2* |
| | C20:5 n-3 | 16.3 ± 16.3 | 20.3 ± 6.1 | 13.4 ± 15.9 | 16.5 ± 16.1 |
| | C22:5 n-3 | N.D. | 3.6 ± 6.2 | N.D. | N.D. |
| | C22:6 n-3 | 31.4 ± 5.6 | 19.0 ± 16.7 | 17.6 ± 5.4 | 19.0 ± 16.5 |

| | | | | | |
|---|---|---|---|---|---|
| | *Σω-3 PUFAs* | *47.8 ± 21.7* | *42.9 ± 25.1* | *31.0 ± 21.3* | *35.6 ± 31.6* |
| | C18:2 n-6 | 60.8 ± 29.8 | 21.8 ± 5.5 | 32.4 ± 7.9 | 18.6 ± 16.2 |
| | C20:4 n-6 | 37.7 ± 34.5 | 57.1 ± 8.7 | 33.9 ± 9.8 | 43.2 ± 37.6 |
| | *Σω-6 PUFAs* | *98.5 ± 30.3* | *79.0 ± 14.2* | *66.3 ± 13.4* | *61.8 ± 53.8* |

**RNA isolation and sequencing**

Total ribonucleic acid (RNA) was isolated from PCATS using RNeasy Lipid Mini Kit (Qiagen, Germantown, MD, United States) according to the manufacturer's protocol. Triplicate samples (~60 mg of PCATS per pup) were pooled and homogenized in 1 mL of Qiazol (Qiagen) using the Bullet Blender Storm 24 (Next Advance, United States; 2 cycles of 2 minutes at 20 mHz) followed by genomic DNA disruption using a 21-gauge needle. After a phase extraction with chloroform, the aqueous phase containing RNA was purified using RNeasy columns with a 20 minute on-column DNase I digest (Qiagen). RNA was eluted in diethylpyrocarbonate (DEPC) water. RNA sample concentration was quantified using RNA High Sensitivity Assay on the Qubit 3.0 fluorometer (Thermo Fisher Scientific) and integrity was assessed using the RNA Pico kit on the 2100 Bioanalyzer (Agilent Technologies). Strand-specific low-input (200 ng) cDNA library preparation and Illumina paired-end MiSeq sequencing were carried out by the DNA Technologies and Expression Analysis Core at the UC Davis Genome Center, supported by NIH Shared Instrumentation Grant 1S10OD010786-01, which produced mean (± s.d.) 34.6 ± 6.2 million reads per sample.

**Transcriptomic analysis**

Transcriptome analyses were conducted using the Bridges 2 high-performance computing cluster at the Pittsburgh Supercomputing Center using Advanced Cyberinfrastructure Coordination

Ecosystem: Services & Support (ACCESS; allocation IBN150010). Sequence quality was assessed using FastQC v0.11.9. The transcriptome was assembled *de novo* using Trinity v2.11.0 with default settings[16]. Transcript redundancy was reduced by clustering using MMSeqs2 (cluster-mode 2, cov-mode 1, coverage threshold 0.95)[17]. Clustering reduced the number of assembled transcripts from 1,629,424 to 1,045,492. The completeness of the clustered transcriptome assembly was assessed using BUSCO v5.0.0 and the Mammalia database of complete Benchmarking Universal Single-Copy Orthologues (BUSCOs), which identified 82.2% complete, 60.6% single-copy, 21.6% duplicated, 6.4% fragmented, and 11.4% missing BUSCOs. Transcript abundance was estimated using Salmon v1.9.0[18]. Transcripts were annotated by BLASTX against the UniProt SwissProt Mammalia and Caniformia databases (downloaded on Nov 7, 2022) using DIAMOND v2.0.13 in ultrasensitive mode with an e-value threshold of 1e-3 and max-target-seqs 1. Differentially expressed transcripts lacking BLASTX hits were annotated by BLASTN against the RefSeq database.

## REFERENCES


1. Louis, C. *et al.* Mobilisation of lipophilic pollutants from blubber in northern elephant seal pups (Mirounga angustirostris) during the post-weaning fast. *Environ. Res.* **132**, 438–448 (2014).

2. Peterson, S. H. *et al.* Effects of Age, Adipose Percent, and Reproduction on PCB Concentrations and Profiles in an Extreme Fasting North Pacific Marine Mammal. *PLoS One* **9**, 96191 (2014).

3. Debier, C. *et al.* Quantitative dynamics of PCB transfer from mother to pup during lactation in UK grey seals Halichoerus grypus. *Mar. Ecol. Prog. Ser.* **247**, 237–248 (2003).

4. Robinson, K. J. *et al.* Investigating decadal changes in persistent organic pollutants in Scottish grey seal pups. *Aquat. Conserv.* **29**, 86–100 (2019).

5. Letcher, R. J. *et al.* Legacy and new halogenated persistent organic pollutants in polar bears from a contamination hotspot in the Arctic, Hudson Bay Canada. *Science of The Total Environment* **610–611**, 121–136 (2018).

6. Brown, T. M., Macdonald, R. W., Muir, D. C. G. & Letcher, R. J. The distribution and trends of persistent organic pollutants and mercury in marine mammals from Canada's Eastern Arctic. *Science of The Total Environment* **618**, 500–517 (2018).

7. Routti, H., Lydersen, C., Hanssen, L. & Kovacs, K. M. Contaminant levels in the world's northernmost harbor seals (Phoca vitulina). *Mar. Pollut. Bull.* **87**, 140–146 (2014).

8. Atkinson, S., Branson, M., Burdin, A., Boyd, D. & Ylitalo, G. M. Persistent organic pollutants in killer whales (Orcinus orca) of the Russian Far East. *Mar. Pollut. Bull.* **149**, 110593 (2019).

9. Panti, C. *et al.* Ecotoxicological Characterization of Type C Killer Whales From Terra Nova Bay (Ross Sea, Antarctica): Molecular Biomarkers, Legacy, and Emerging Persistent Organic Contaminants. *Front. Mar. Sci.* **9**, 818370 (2022).

10. Wolkers, H., Corkeron, P. J., Van Parijs, S. M., Similä, T. & Van Bavel, B. Accumulation and transfer of contaminants in killer whales (Orcinus orca) from Norway: Indications for contaminant metabolism. *Environ. Toxicol. Chem.* **26**, 1582–1590 (2007).

11. Vetter, W., Lok, B., Schlienz, A. & Müller, M. Thorough study of persistent organic pollutants and halogenated natural products in sperm whale blubber through preparative sample cleanup followed by fractionation with countercurrent chromatography. *J. Chromatogr. A* **1565**, 114–123 (2018).

12. Miranda Filho, K. C. *et al.* Lactational transfer of PCBs and chlorinated pesticides in pups of southern elephant seals (Mirounga leonina) from Antarctica. *Chemosphere* **75**, 610–616 (2009).

13. Lawson, T. M. *et al.* Concentrations and profiles of organochlorine contaminants in North Pacific resident and transient killer whale (Orcinus orca) populations. *Science of The Total Environment* **722**, 137776 (2020).

14. Beltest I014-Rev4-17/7/2000-17. Determination of polychlorinated biphenyls in animal feed, animal fat, egg and egg products, milk products and other foodstuffs. Preprint at (2000).

15. Bligh, E. G. & Dyer, W. J. A rapid method of total lipid extraction and purification. *Can. J. Biochem. Physiol.* **37**, 911–917 (1959).

16. Haas, B. J. *et al.* De novo transcript sequence reconstruction from RNA-seq using the Trinity platform for reference generation and analysis. *Nature Protocols 2013 8:8* **8**, 1494–1512 (2013).

17. Steinegger, M. & Söding, J. Clustering huge protein sequence sets in linear time. *Nature Communications 2018 9:1* **9**, 1–8 (2018).

18. Patro, R., Duggal, G., Love, M. I., Irizarry, R. A. & Kingsford, C. Salmon provides fast and bias-aware quantification of transcript expression. *Nature Methods 2017 14:4* **14**, 417–419 (2017).
